# β_1_-Adrenergic Receptor Recycles Via a Membranous Organelle, Recycling Endosome, by Binding with Sorting Nexin27

**DOI:** 10.1007/s00232-013-9571-6

**Published:** 2013-06-19

**Authors:** Takatoshi Nakagawa, Michio Asahi

**Affiliations:** Department of Pharmacology, Faculty of Medicine, Osaka Medical College, 2-7 Daigaku-machi, Takatsuki, Osaka 569-8686 Japan

**Keywords:** β_1_-Adrenergic receptor, Desensitization, Recycling endosome, SNX27

## Abstract

In cardiomyocytes, β_1_-adrenergic receptor (β_1_-AR) plays an important role in regulating cardiac functions. Upon continuous ligand stimulation, β_1_-AR is internalized and mostly recycled back to the plasma membrane (PM). The recycling endosome (RE) is one of the membranous organelles involved in the protein recycling pathway. To determine whether RE is involved in the internalization of β_1_-AR upon ligand stimulation, we evaluated the localization of β_1_-AR after stimulation with a β-agonist, isoproterenol (Iso), in β_1_-AR-transfected COS-1 cells. After 30 min of Iso treatment and cell surface labeling with the appropriate antibodies, β_1_-AR was internalized from PM and translocated into the perinuclear region, the same location as the transferrin receptor, an RE marker. We then evaluated whether sorting nexin 27 (SNX27) participated in the β_1_-AR recycling pathway. When β_1_-AR and SNX27 were coexpressed, β_1_-AR coimmunoprecipitated with SNX27. In addition, shRNA-mediated silencing of SNX27 compromised β_1_-AR recycling and enhanced its delivery into lysosome. Overall, β_1_-AR on PM was internalized into RE upon Iso stimulation and recycled by RE through binding with SNX27 in COS-1 cells.

## Introduction

β-Adrenergic receptors (β-ARs) are trimeric G protein-coupled receptors that mediate physiological responses to epinephrine and norepinephrine (Strader et al. 1995). β_1_- and β_2_-ARs are expressed in the cardiac system and have been shown to play different roles in cardiac function and development (Rohrer et al. 1996). The major subtype of ARs expressed in cardiomyocytes is β_1_-AR, whereas β_2_-AR expression is minor (Engelhardt et al. 1999). Upon ligand engagement, β_2_-AR is reported to be internalized, sorted into early endosome and quickly recycled back into the plasma membrane (PM) through recycling endosome (RE) (Parent et al. 2009; Seachrist et al. 2000). Stimulation by the ligand activates GRK2, which in turn phosphorylates β-ARs and creates docking sites for β-arrestins (β-Arrs). The binding of β-Arrs to the activated β-ARs triggers receptor internalization (Krasel et al. 2005). In transiently transfected HEK293 cells, exposure to an agonist for a short time leads to the phosphorylation of β_1_-AR by cAMP-dependent protein kinase and GRKs, decreasing the response to stimulation by a second agonist. In addition, overexpression of β-Arr1 and β-Arr2 enhances the uncoupling of β_1_-AR from G proteins. These results indicate that β-Arrs are involved in the uncoupling of β_1_-AR as well as β_2_-AR (Freedman et al. 1995). Interestingly, the ligand-induced internalization of β_2_-AR has been observed to be quite extensive, whereas β_1_-AR is internalized to a lesser extent (Liang et al. 2004).

The accurate sorting of proteins to proper organelles is essential to many cellular activities. Internalized receptors are sorted to different organelles through endocytic membrane trafficking (Mellman 1996). Receptors that are being recycled are separated from those bound for degradation and either directly recycled to the PM or transported to the perinuclear RE (Yamashiro et al. 1984). Recent findings revealed that many important molecules transiently or steadily reside in RE (Misaki et al. 2010). The internalization and translocation of β_2_-AR into RE are controlled by Rab11 (Parent et al. 2009). However, little is known about β_1_-AR.

To determine the localization of molecules in specific organelles, it is important to choose an appropriate cell system that has high spatial resolution of organelles in perinuclear regions [early endosome (EE), RE and Golgi]. To determine the localization of β_1_-AR in dormant and activated states, we used COS-1 cells. Because of the unique spatial organization of organelles in COS-1 cells, RE is exclusively confined within the ring-shaped structure of the Golgi (Golgi ring) and the organelles associated with degradation [EE, late endosome (LE) and lysosome] are excluded from inside the Golgi ring (Misaki et al. 2007).

Sorting nexins (SNXs) are proteins characterized by the presence of the phox-homology (PX) domain, which binds to phosphatidylinositol-3-monophosphate (PI-3P). SNXs are conserved among a wide variety of species; thus far, more than 30 SNXs have been identified in mammals (Cullen 2008). Through the interaction of the SNX PX domain with PI-3P on the membrane, these proteins participate in various processes including endocytosis, endosomal sorting and endosomal signaling.

Among SNXs, SNX27 is unique in that it has a PDZ (postsynaptic density protein-95, Discs-large, Zona-occludens-1) domain, which functions as a scaffold to organize various proteins. Efficient recycling of β_2_-AR is dependent on its C-terminal PDZ-domain binding motif (Cao et al. 1999). Recently, SNX27 has been reported to be a prerequisite for efficient PDZ-directed recycling of various receptors such as β_2_-AR, *N*-methyl-d-aspartate receptor 2C or glutamate receptors from EE to PM (Lauffer et al. 2010; Cai et al. 2011; Wang et al. 2013). SNX27 might be involved in the functional regulation of many proteins with PDZ-domain binding motifs (Cai et al. 2011; Wang et al. 2013). Furthermore, it was shown that SNX27 mediates the recycling of PDZ-domain binding motif-containing cargo, β_2_-AR, by linking to the retromer, a complex of proteins that has been shown to be important in recycling (Temkin et al. 2011). A large number of proteins were shown to be recycled by an SNX27/retromer complex (Steinberg et al. 2013).

Using COS-1 cells, we clearly demonstrated that β_1_-AR localizes mainly at PM and the β-agonist isoproterenol (Iso) triggered the internalization of β_1_-AR from PM and its translocation into RE. Furthermore, we observed that the recycling of β_1_-AR after Iso-induced internalization is regulated by its binding with SNX27.

## Materials and Methods

### Cell Culture

COS-1 cells were maintained in DMEM supplemented with 10 % fetal bovine serum, 100 U/ml penicillin and 100 μg/ml streptomycin, as described previously (Misaki et al. 2007).

### Cloning of β_1_-AR and SNX27

β_1_-AR and SNX27 were cloned from mouse heart using a conventional RT-PCR method. In brief, mRNA was extracted from mouse heart using Isogen II (Nippongene, Tokyo, Japan), and the cDNA was synthesized using superscript III (Invitrogen, Carlsbad, CA) according to the manufacturer’s instructions. Using the synthesized cDNA as a template, PCRs were performed using PrimeStar (Takara-bio, Shiga, Japan) with the following primers: β_1_-AR, 5′-CGAGGGATCCCTCGGCATGGGCGCGGGGGCGCTCG-3′ (forward primer), 5′-GGTGCTCGAGCTACACCTTGGACTCCGAGGAGAAGC-3′ (reverse primer) and SNX27, 5′-GAGCAAGCTTGCTCGCAAGATGGCGGAC-3′ (forward primer), 5′-CACCGTCGACGGTGGCCACATCCCTCTGCTG-3′ (reverse primer). Amplified fragments for β_1_-AR and SNX27 were subcloned into pcDNA3.1Myc.His (Invitrogen) at the *Bam*H1/*Xho*I or *Hin*dIII/*Sal*I site, respectively. The resulting constructs were confirmed by DNA sequencing. β_1_-AR (HA-tagged) was constructed using PCR-based insertion of the HA-tag sequence into pcDNA3.1Myc.His/β_1_-AR at the amino-terminal using PrimeStar (Takara-bio). The primers used were as follows: 5′-GTTCCAGATTACGCTGGCGCGGGGGCGCTCGCCCTG-3′ (forward primer) and 5′-ATCGTATGGGTACATGCCGAGGGATCCGAGCTC-3′ (reverse primer).

To establish COS-1 cells that stably express β_1_-AR (HA-tagged), the DNA encoding β_1_-AR (HA-tagged) was subcloned into the pLHCX vector (Clontech, Mountain View, CA) at the *Hin*dIII/*Xho*I site using the following primers: 5′-CGAGAAGCTTCTCGGCATGTACCCATACGATG-3′ (forward primer) and 5′-GTAGATCGATCTACACCTTGGACTCCGAGG-3′ (reverse primer), pLHCX/β_1_-AR. To make a mutant β_1_-AR that lacks last four amino acids at the carboxy terminus (∆C4β_1_-AR), we mutated a pLHCX/β_1_-AR using a PCR with the following primers: 5′-CGGCCCCGCGGTCCCGAAGAGGAGCATCTAGCTA-3′ (forward primer) and 5′-GTAGATCGATCTACGAGGAGAAGCCCTGGCGCC-3′. A recombinant retrovirus harboring β_1_-AR (HA-tagged) was prepared using the Retrovirus Packaging kit (Ampho) (Takara-bio) according to the manufacturer’s instructions. The pLHCX/β_1_-AR (HA-tagged) retrovirus was infected into COS-1 cells, and the infected COS-1 cells were selected by growing in 200 μg/ml hygromycin (Wako, Osaka, Japan) for approximately 2 weeks. After evaluating the expression level of β_1_-AR in the surviving cells, this cell line was used for the experiments. We named the COS-1 cells stably expressing β_1_-AR (HA-tagged) as “β_1_-AR cells.”

### Antibodies

We used the following polyclonal antibodies (Abs) in this study: anti-β_1_-AR Ab (Santa Cruz Biotechnology, Santa Cruz, CA); anti-HA Ab and anti-EEA1 Ab (MBL, Aichi, Japan). In addition, we used several monoclonal Abs: anti-human TfnR Ab (Invitrogen), anti-GM130 Ab (BD Bioscience, Rockville, MD), anti-LAMP1 Ab (Santa Cruz Biotechnology) and anti-Myc Ab (9E10; Biomol, Hamburg, Germany).

### Immunostaining

COS-1 cells transfected with the plasmids of interest or β_1_-AR cells were plated on cover glasses in a 24-well plate. After treatment with 50 μg/ml cycloheximide for 4 h, cells were fixed for 10 min, permeabilized for 10 min and blocked in 4 % paraformaldehyde, 0.1 % Triton X-100 and 5 % bovine serum albumin (BSA) for 30 min at room temperature. The primary Ab diluted 1:1,000 with 5 % BSA was added and incubated for 30 min at room temperature. After washing with phosphate-buffered saline (PBS) thrice, the secondary Ab (Alexa546-labeled anti-rabbit Ab for the anti-HA Ab or Alexa488-labeled anti-mouse Ab for the anti-GM130 and anti-TfnR Ab) was added. To track the internalization of β_1_-AR from the cell surface, Iso and the primary Ab were added simultaneously before fixation.

### Confocal Microscopy

For confocal microscopy, we used LSM500 (Carl Zeiss, Oberkochen, Germany). Images of the fixed cells were taken at ambient temperature with a 63 × 1.4 NA Plan Apochromat oil-immersion lens. Excitations were performed using two lasers, one emitting at 488 nm and the other at 543 nm. Emissions were collected using appropriate filters.

### Cell Surface Biotinylation

Two types of cell surface biotinylation experiments were performed upon Iso treatment; one was designed to evaluate the reduction in β_1_-AR on the cell surface, whereas the other was designed to evaluate the degradation of β_1_-AR on the cell surface.

#### Degradation Rate of Cell Surface β_1_-AR

Cells were biotinylated preceding Iso treatment. Biotinylated cells were quenched with 0.1 M glycine, washed several times with prewarmed DMEM and then further incubated for 4 h in the presence of 10^−6^ M Iso. Cells were washed with PBS thrice and lysed with RIPA buffer (25 mM Tris, 0.14 M NaCl, 1 % NP40, 0.5 % deoxycholic acid, pH 8.0). Biotinylated proteins were precipitated with avidin-agarose (Pierce, Rockford, IL) and eluted with 1× SDS sample buffer (0.125 M Tris, 2 % SDS, 10 % glycerol, pH 6.8). Eluates were subjected to SDS-PAGE and Western blot analysis.

#### Reduction Rate of Cell Surface β_1_-AR

COS-1 cells transiently expressing β_1_-AR or β_1_-AR cells were starved in serum-free DMEM for 2 h and incubated for 30 min with or without 10^−6^ M Iso. To evaluate the recovery of once-internalized receptors, cells were further incubated for 60 min after the removal of Iso. Cells were then washed with PBS thrice and biotinylated with 0.1 mg/ml sulfo-NHS-biotin (Pierce) in PBS with 1 mM CaCl_2_ and 0.5 mM MgCl_2_ for 30 min. After 30 min, cells were quenched with 0.1 M glycine. The preceding steps were mentioned in the previous section.

### Western Blot Analysis

Following SDS-PAGE, the gel was put into a semidry blotting system (Bio-Rad, Hercules, CA) and proteins were transferred onto a PVDF membrane (PALL, Port Washington, NY). The membrane was blocked with 5 % skimmed milk in PBS containing 0.1 % Tween 20 (PBST, blocking buffer), incubated with primary Ab of interest in blocking buffer, washed thrice with PBST and then incubated with the secondary Ab conjugated with horseradish peroxidase. The membrane was visualized with Immobilon Western (Millipore, Hayward, CA), and the image was captured with Chemidoc (Bio-Rad).

### Coimmunoprecipitation of β_1_-AR or ∆C4β_1_-AR with SNX27

β_1_-AR or ∆C4β_1_-AR was cotransfected with SNX27 (Myc-tagged) in COS-1 cells. After 2 h of starvation in serum-free DMEM, transfected cells were incubated with or without 10^−6^ M Iso for 10 min. Cells were then collected, washed thrice with PBS and lysed with lysis buffer (10 mM Tris, 50 mM NaCl, 1 % NP40) containing a protease inhibitor cocktail (Nacalai Tesque, Kyoto, Japan). Lysates were incubated with protein G agarose premixed with an anti-Myc Ab for several hours; the protein G agarose was then washed thrice with lysis buffer. Precipitated proteins were eluted in the same volume of 2× SDS sample buffer by boiling for 2 min and subjected to SDS-PAGE, followed by Western blot analysis as mentioned previously.

### SNX27 Silencing Using shRNA

shRNA against SNX27, targeting the sequence AAGGACCATAGACAGAGATTA (shSNX27), was cloned into the pSIN vector (Takara-bio) according to the manufacturer’s instructions. shSNX27 was transfected into β_1_-AR cells using Lipofectamine 2000 (Invitrogen). In brief, 2 μg of shSNX27 in 100 μl of Opti-MEM was mixed with 5 μl of Lipofectamine 2000 diluted in 100 μl of Opti-MEM. The mixture was incubated at room temperature for 20 min and then added to cells that were cultured overnight in 1 ml of antibiotic-free medium in 12-well plates. After 48 h, cells were used for the experiments including Western blot analysis, real-time PCR, cell surface biotinylation, coimmunoprecipitation and immunostaining.

### Real-Time Reverse Transcription PCR

Total RNAs were prepared using a TRIzol reagent (Invitrogen), according to the manufacturer’s instructions. Real-time RT-PCR analyses were performed using a Thermal Cycler Dice Real Time System Single MRQ (Takara-bio). cDNA was synthesized using a PrimeScript RT reagent kit (Takara-bio), and PCRs were performed using SYPR premix ExTaq II (Takara-bio), according to the manufacturer’s instructions. The mean number of cycles required to reach the threshold of fluorescence detection (Ct value) was calculated for each sample, and glyceraldehyde 3-phosphate dehydrogenase (GAPDH) expression was quantified for normalization of the amount of cDNA in each sample. The specificity of the amplified products was evaluated by the melting curve. The primer sets used to quantitate SNX27 and GAPDH, respectively, were as follows: 5′-ACCACCTGCTTGTGTGTCG-3′ (forward) and 5′-ATCAACGGGGAGCTGTACG-3′ (reverse) and 5′-ATTGCCCTCAACGACCACTT-3′ (forward) and 5′-AGGTCCACCACCCTGTTGCT-3′ (reverse).

## Results and Discussion

### Cellular Localization of β_1_-AR at Steady State

**Fig. 1:**
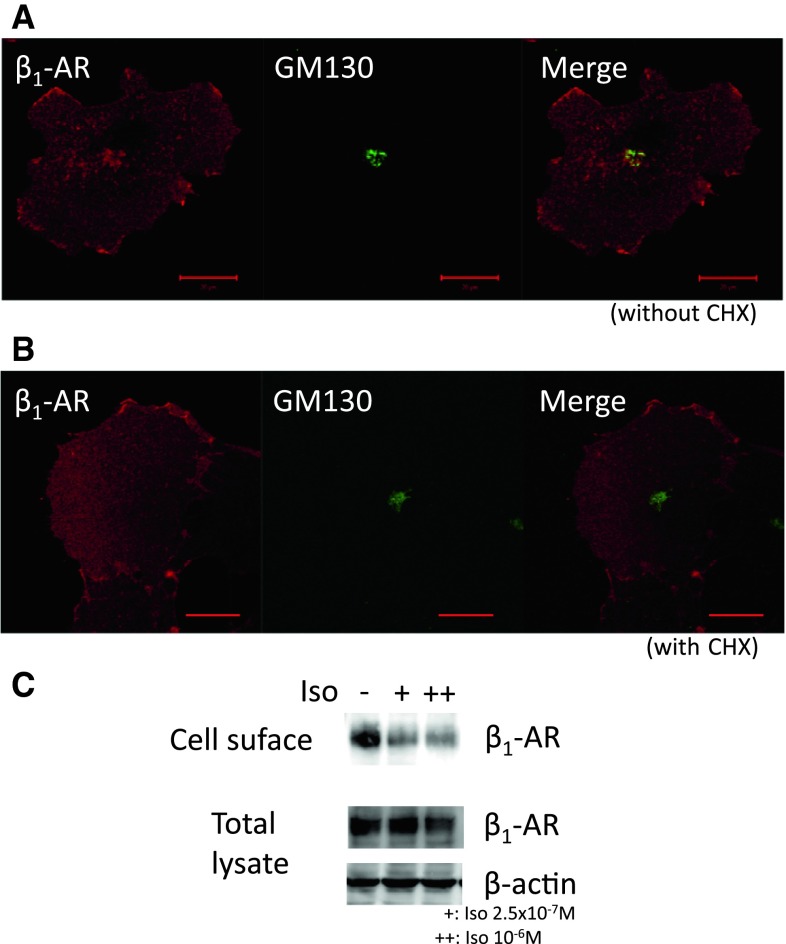
Steady-state localization and internalization of β_1_-AR upon Iso stimulation in COS-1 cells. β_1_-AR cells were treated without (**a**) or with (**b**) cycloheximide (*CHX*, 50 μg/ml) for 4 h and stained with anti-HA or anti-GM130 Abs. Images were captured using the LSM500. **c** Internalization of β_1_-AR from the cell surface. β_1_-AR cells were biotinylated using 0.1 mg/ml NHS-sulfo-biotin on ice for 30 min, quenched with 0.1 M glycine, washed with prewarmed DMEM thrice and then incubated with 2.5 × 10^−7^ (+) or 10^−6^ M (++) of Iso for 2 h. Biotinylated proteins concentrated with avidin-agarose were subjected to SDS-PAGE followed by Western blotting (*upper panel* cell surface proteins probed with anti-β_1_-AR Ab; *middle and lower panels* total proteins probed with anti-β_1_-AR Ab or anti-β-actin Ab, respectively). *Bar* 20 μm

In COS-1 cells, the organelles involved in internalization, such as EE, LE, RE and Golgi, can be visually segregated (Misaki et al. 2007). In this respect, COS-1 cells are suitable for elucidation of intracellular trafficking of β_1_-AR. To determine the distribution of β_1_-AR at steady state in COS-1 cells, we established cells stably expressing β_1_-AR proteins that were N-terminally tagged with the HA epitope (β_1_-AR cells). First, the costaining of β_1_-AR with GM130, a *cis*-Golgi marker, in β_1_-AR cells was performed. The results show that β_1_-AR was expressed not only on the cell surface but also in the Golgi (Fig. 1a). Both β_1_-AR and GM130 formed typical “Golgi-ring” structures. The ring-shaped Golgi is characteristic of COS-1 cells. However, when cells were treated with the protein synthesis inhibitor cycloheximide for 4 h before immunostaining, the ring-shaped Golgi structure visualized by β_1_-AR staining had disappeared (Fig. 1b). This result indicated that β_1_-AR was exclusively expressed on the cell surface at steady state in COS-1 cells.

### Internalization of β_1_-AR Upon Ligand Stimulation

β-ARs are known to be internalized upon continuous ligand stimulation. To reveal how β_1_-AR on the cell surface is internalized, we attempted to track the behavior of β_1_-AR upon ligand stimulation in COS-1 cells by performing a cell surface biotinylation experiment. By comparing the amounts of β_1_-AR on the cell surface with or without Iso treatment, we were able to biochemically verify whether β_1_-AR was internalized with Iso. COS-1 cells expressing β_1_-AR were stimulated with Iso after biotinylation of the cell surface. Biotinylated proteins were subjected to SDS-PAGE followed by Western blot analysis. As shown in Fig. 1c, the total amount of β_1_-AR remained unchanged after stimulation with Iso. In contrast, β_1_-AR on the cell surface decreased significantly after Iso treatment. We concluded that β_1_-AR is internalized upon ligand stimulation in COS-1 cells.

### Translocation of Internalized β_1_-AR to RE

**Fig. 2:**
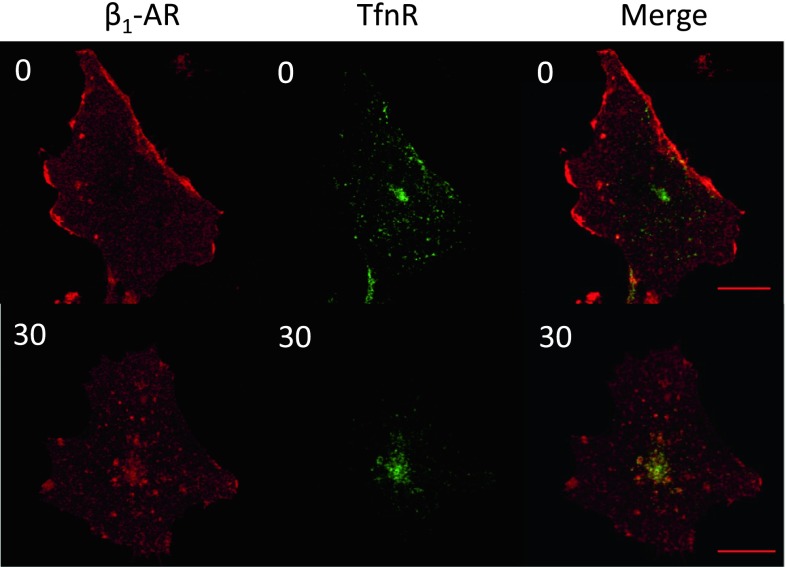
Effects of Iso stimulation on the internalization of β_1_-AR into RE in COS-1 cells. β_1_-AR cells were plated onto glass coverslips. Cells were incubated with or without 10^−6^ M Iso for 30 min in the presence of anti-HA Ab. Cells were then fixed with 4 % paraformaldehyde followed by 0.1 % Triton X-100 (for 10 min in each step). Anti-TfnR Ab was added and incubated for 1 h at room temperature. Abs against HA or TfnR were probed with Alexa546-labeled anti-rabbit or Alexa488-labeled anti-mouse Abs, respectively. A laser scanning microscope (LSM500) was used to capture the images (*upper panel* images without Iso; *lower panel* images with Iso for 30 min)

We monitored the internalized β_1_-AR from the cell surface after 30 min of Iso treatment. The internalized β_1_-AR was costained with an Ab against the transferrin receptor (TfnR), which is localized predominantly in RE. RE is involved not only in the transit of recycling molecules, such as TfnR or LDL receptor but also in various biological activities including anterograde transport (Ang et al. 2004) and polarized localization of E-cadherin (Lock and Stow 2005). We examined whether β_1_-AR localizes to RE, the TfnR-positive organelle, after ligand stimulation. As shown in Fig. 2, strong signals of β_1_-AR were observed around PM, and no discrete intracellular structures were present. In contrast, TfnR resided exclusively in the perinuclear region, showing localization patterns typical of proteins that are located in RE (Fig. 2). No overlapping was observed between the two proteins without ligand stimulation (at 0 min). Iso treatment induced drastic changes in β_1_-AR localization. Strong β_1_-AR signals appeared in the perinuclear region, which was well merged with TfnR, clearly indicating that β_1_-AR was translocated from the cell surface to RE upon ligand stimulation.

Intriguingly, small G proteins, such as ras (Misaki et al. 2010) and rap2 (Uechi et al. 2009), are located in RE, which seems to be critical for their functions. Palmitoylation is quite important for ras and rap2 localization to RE, and a recent study reported that β_1_-AR is also palmitoylated (Zuckerman et al. 2011). RE is not only a simple receptacle for recycled molecules but also a dynamic organelle where many signaling molecules are localized. β_1_-AR may be modified or bound to signaling molecules through RE, resulting in other cellular functions.

### Recycling of β_1_-AR Back to PM

**Fig. 3:**
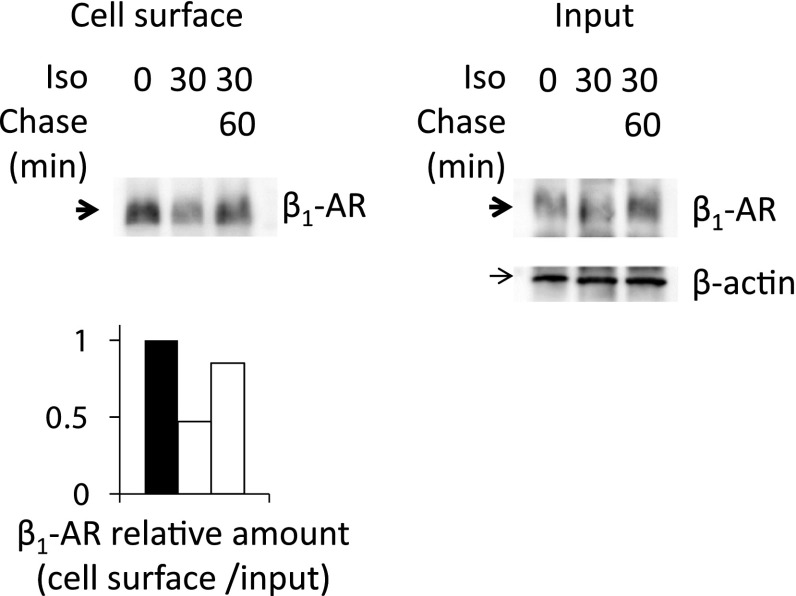
Recycling of internalized β_1_-AR to the cell surface after Iso stimulation in COS-1 cells. Cells expressing β_1_-AR were incubated with 10^−6^ M Iso. After 30 min of incubation, Iso was removed and the cells were further incubated for 60 min. To quantify the amount of β_1_-AR on the cell surface, cells were then biotinylated. Biotinylated proteins were subjected to SDS-PAGE and Western blot analyses. Cell surface biotinylated proteins probed with anti-β_1_-AR Ab; *Input* total proteins probed with β_1_-AR (*upper*) or β-actin Ab (*lower*). *Lower panel* Quantitative analyses of β_1_-AR on the cell surface relative to total β_1_-AR were densitometrically analyzed using Quantity One (Bio-Rad). The ratio of β_1_-AR on the cell surface relative to total β_1_-AR normalized with β-actin is shown. Most of the β_1_-AR that was once internalized by Iso treatment was recovered to the cell surface at 60 min after the removal of Iso (*arrow*)

Since TfnR and the LDL receptor have been shown to be recycled through RE upon continuous ligand stimulation (Ang et al. 2004), we examined whether β_1_-AR could be recycled through RE in COS-1 cells. To confirm the recycling of internalized β_1_-AR biochemically, we compared the amount of biotinylated β_1_-AR on the cell surface before and 30 min after Iso treatment. After Iso removal, cells were cultured for a further 60 min (60 min of chase) (Fig. 3). The results showed that the amount of β_1_-AR on the cell surface decreased significantly after 30 min of Iso treatment and the internalized β_1_-AR was almost completely recovered to the cell surface after 60 min of chase. These results imply that β_1_-AR is recycled by RE after ligand stimulation in COS-1 cells.

### Physical Interaction of SNX27 with β_1_-AR in EE

**Fig. 4:**
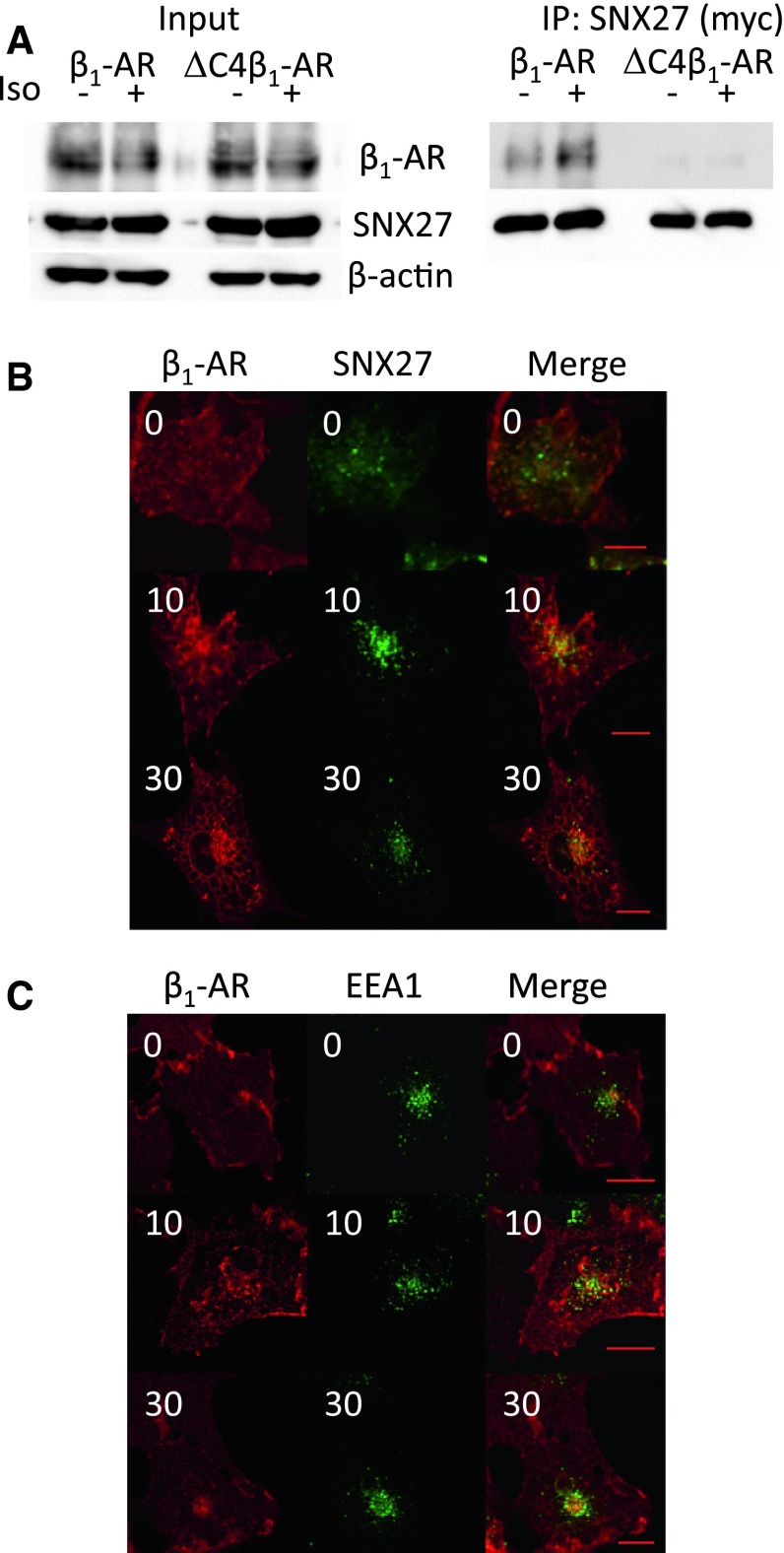
Role of SNX27 in β_1_-AR recycling after Iso stimulation in COS-1 cells. Coimmunoprecipitation of β_1_-AR or ∆C4β_1_-AR with SNX27. COS-1 cells were transfected with β_1_-AR or ∆C4β_1_-AR and SNX27 (myc-tagged). Cells were stimulated with or without 10^−6^ M Iso for 10 min, lysed and coimmunoprecipitated with anti-Myc Ab. Precipitated proteins were separated by SDS-PAGE and immunoblotted with anti-β_1_-AR or Myc Abs. Lysates (*Input*) were also probed with the same Abs as well as anti-β-actin Ab. Localization of β_1_-AR and SNX27 (**b**) or EE (**c**). β_1_-AR was transfected into COS-1 cells with or without SNX27 (**b**, **c**, respectively). Transfected cells were plated onto glass coverslips, treated with 10^−6^ M Iso for 10 and 30 min in the presence of anti-HA Ab and coimmunostained with anti-Myc Ab (**b**) or anti-EEA1 Ab (**c**) as described in “Materials and Methods” images were taken with the LSM500 microscope. *Bar* 20 μm

SNX27 has been shown to play an important role in β_2_-AR endosomal trafficking, particularly recycling (Lauffer et al. 2010). Since β_1_-AR has a class I PDZ-binding motif with a noncharged amino acid at −5 position (Balana et al. 2010), we hypothesized that SNX27 participates in the β_1_-AR membrane trafficking pathway as well. First, we addressed whether β_1_-AR can physically interact with SNX27. COS-1 cells were transfected with β_1_-AR (HA-tagged) and SNX27 (Myc-tagged). Cell lysates were coimmunoprecipitated with anti-Myc Ab followed by probing with anti-β_1_-AR or anti-Myc Ab. As shown in Fig. 4a, β_1_-AR was coimmunoprecipitated with anti-Myc Ab. The interaction was augmented by 10 min of stimulation with Iso, suggesting that β_1_-AR can physically interact with SNX27. The interaction of β_2_-AR with SNX27 was reported to be mediated by a PDZ domain-binding motif of β_2_-AR (Lauffer et al. 2010). As expected, ∆C4β_1_-AR, which is a mutant β_1_-AR that lacks four amino acids at the carboxy terminus that constitute a functional PDZ-binding motif (Hu et al. 2000; Xu et al. 2001), was not coimmunoprecipitated with SNX27, indicating that the interaction of β_1_-AR with SNX27 is also mediated by a PDZ domain–binding motif of β_1_-AR (Fig. 4a).

Next, the subcellular localization of both proteins, particularly in the endocytic pathway, was investigated (Fig. 4b). β_1_-AR was labeled with an anti-HA Ab and observed for localization after stimulation. At 0 min, β_1_-AR was localized on the cell surface. SNX27 was observed in punctate structures in the perinuclear region, as reported for other proteins localized in EE (Lauffer et al. 2010). After 10 min of Iso treatment, the localization of β_1_-AR overlapped with that of SNX27, which is located in EE, indicating that β_1_-AR is colocalized with SNX27 in EE. This observation is consistent with results indicating that β_1_-AR colocalized with EEA1, an EE marker protein (Fig. 4c). Although a fraction of β_1_-AR was still colocalized with SNX27 at 30 min (Fig. 4b), most β_1_-ARs had segregated into a more confined area in the perinuclear region (Fig. 4c).

### Functional Interaction of β_1_-AR with SNX27

**Fig. 5:**
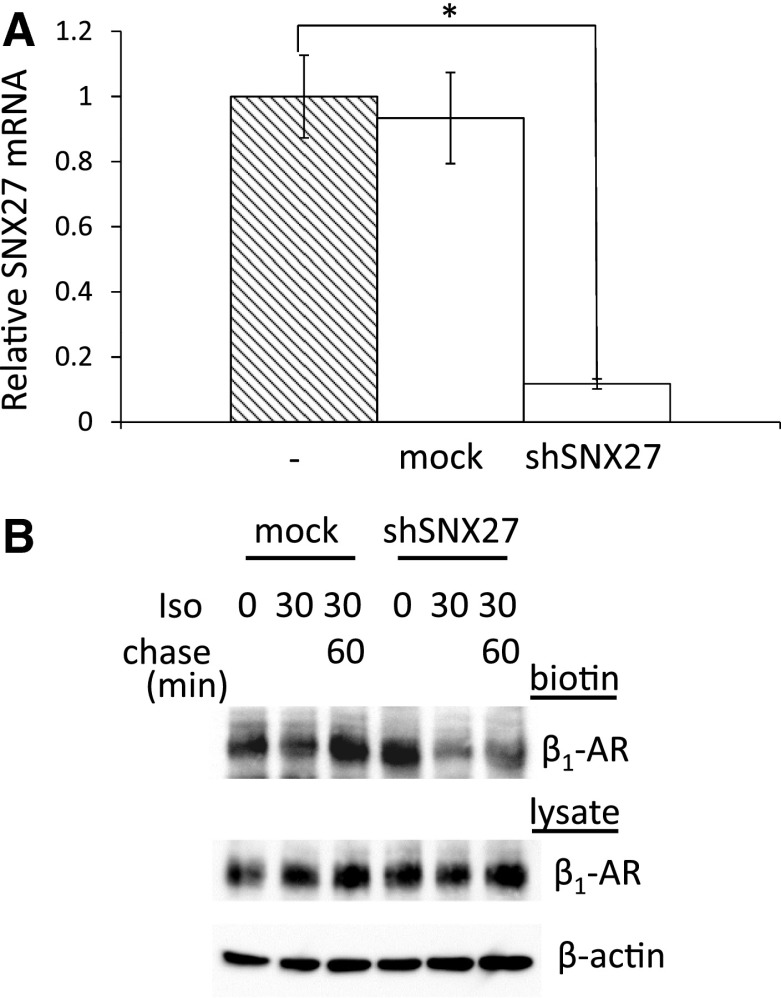
Effects of SNX27 silencing on β_1_-AR recycling after Iso stimulation in COS-1 cells. **a** Evaluation of shSNX27 in COS-1 cells. shSNX27 was transfected into COS-1 cells, and 48 h later, total cellular RNA was extracted, reverse-transcribed and subjected to real-time RT-PCR analysis. SNX27 expression levels in transfected cells were compared with those in nontransfected and empty vector–transfected COS-1 cells. **b** Effects of SNX27 silencing on β_1_-AR recycling. β_1_-AR cells were transfected with shSNX27 or an empty vector (mock), and 48 h later, cells were treated without or with 10^−6^ M Iso for 30 min (0 and 30, respectively). After incubation, cells were washed to remove Iso and further incubated for 60 min (chase) (*Iso 30*, *Chase 60*). Cells were then biotinylated and lysed. Biotinylated proteins were collected with avidin-agarose and subjected to SDS-PAGE, followed by Western blot analysis using anti-β_1_-AR and β-actin Abs. The *p* value was calculated using a Dunnett’s test. *p < 0.05

**Fig. 6:**
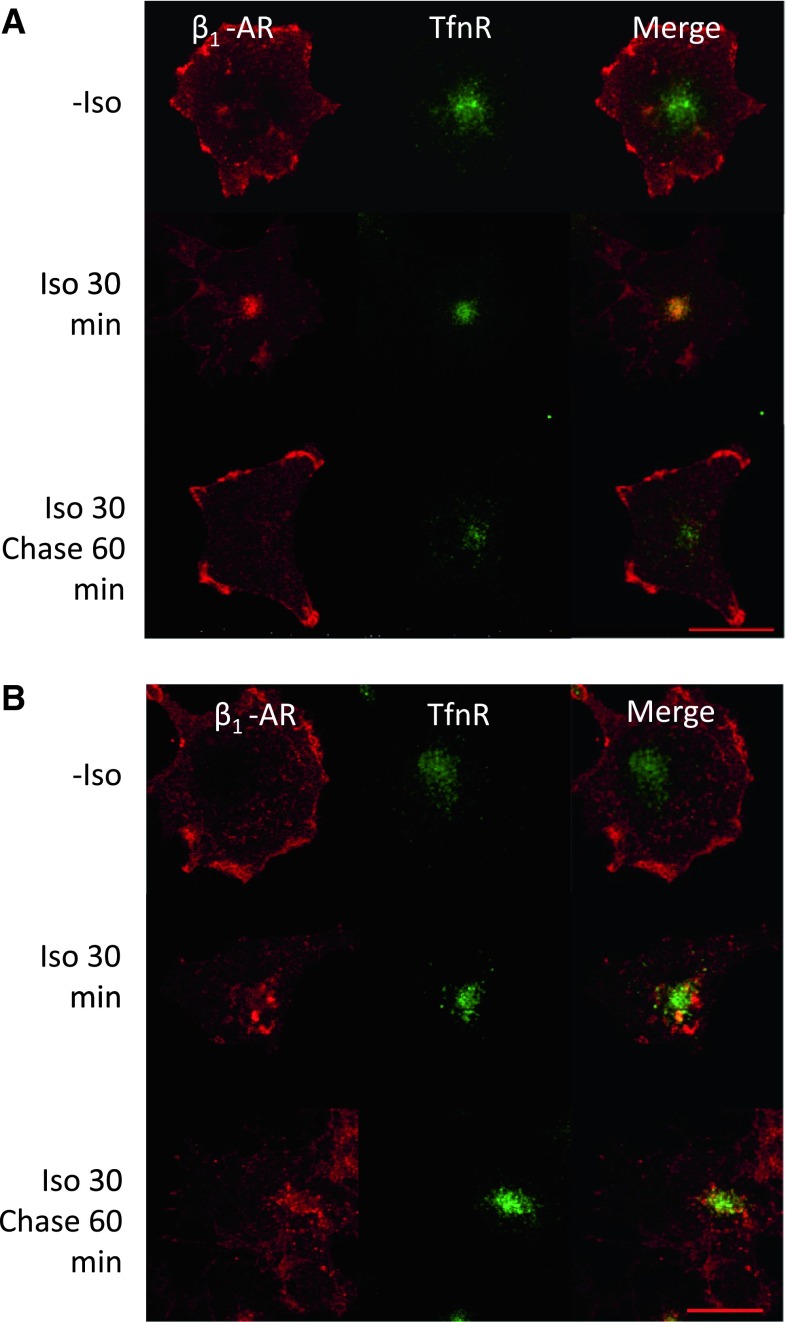
Effects of SNX27 silencing on the subcellular localization of β_1_-AR after Iso stimulation in COS-1 cells. Either empty vector (**a**) or shSNX27 (**b**) was transfected into β_1_-AR cells, and 24 h later, cells were plated onto glass coverslips. After 48 h, cells were treated without or with 10^−6^ M Iso for 30 min (−*Iso* and *Iso 30* *min*, respectively). After incubation, cells were washed to remove Iso and further incubated for 60 min (chase) (*Iso 30* and *Chase 60* *min*) to follow the cell surface recovery of β_1_-AR. During Iso incubation, anti-HA Abs were added to label cell surface proteins. After incubation, cells were fixed and the internalized β_1_-AR (labeled with anti-HA Ab) was costained with anti-TfnR Ab. *Bar* 20 μm

Since β_1_-AR can physically interact with SNX27 at EE (Fig. 4), we next addressed how this physical interaction is involved in the β_1_-AR endocytic pathway. To this end, we silenced SNX27 using an shRNA against SNX27 (shSNX27). Transient transfection of shSNX27 in COS-1 cells diminished the expression of SNX27 by 90 % at the mRNA level (Fig. 5a). As shown in Fig. 3, β_1_-AR on the cell surface was internalized after 30 min of Iso treatment and recycled back to the cell surface after 60 min of chase following Iso removal. To elucidate the functional roles of SNX27 in β_1_-AR recycling, we performed the same experiment using shSNX27. In mock-transfected β_1_-AR cells, β_1_-AR was diminished after 30 min of Iso treatment, whereas during the 60-min chase time, a considerable amount of β_1_-AR was recovered on the cell surface compared with the amount of β_1_-AR on the cell surface at 0 min (Fig. 5b). These data are consistent with those shown in Fig. 3. While the reduction in cell surface β_1_-AR after 30 min of Iso treatment was equivalent in β_1_-AR cells with shSNX27 and mock-transfected β_1_-AR cells, a lesser amount of β_1_-AR was recovered after 60 min of chase in shSNX27-transfected cells. These results suggest that shSNX27 inhibited recycling of the internalized β_1_-AR. To confirm this result, we performed confocal microscopic analysis of cells with or without shSNX27. As shown in Fig. 6, after 30 min of Iso treatment, the β_1_-AR on the cell surface had become internalized and was eventually confined to RE corresponding with the localization of TfnR in mock-transfected cells. After 60 min of chase, the internalized β_1_-AR was recycled back. In contrast, in cells with shSNX27, β_1_-AR expressed on the cell surface became internalized after 30 min of Iso treatment, most of which was segregated from the TfnR-positive RE. After 60 min of chase, β_1_-AR was still inside the cells, in which TfnR and β_1_-AR did not merge.

**Fig. 7:**
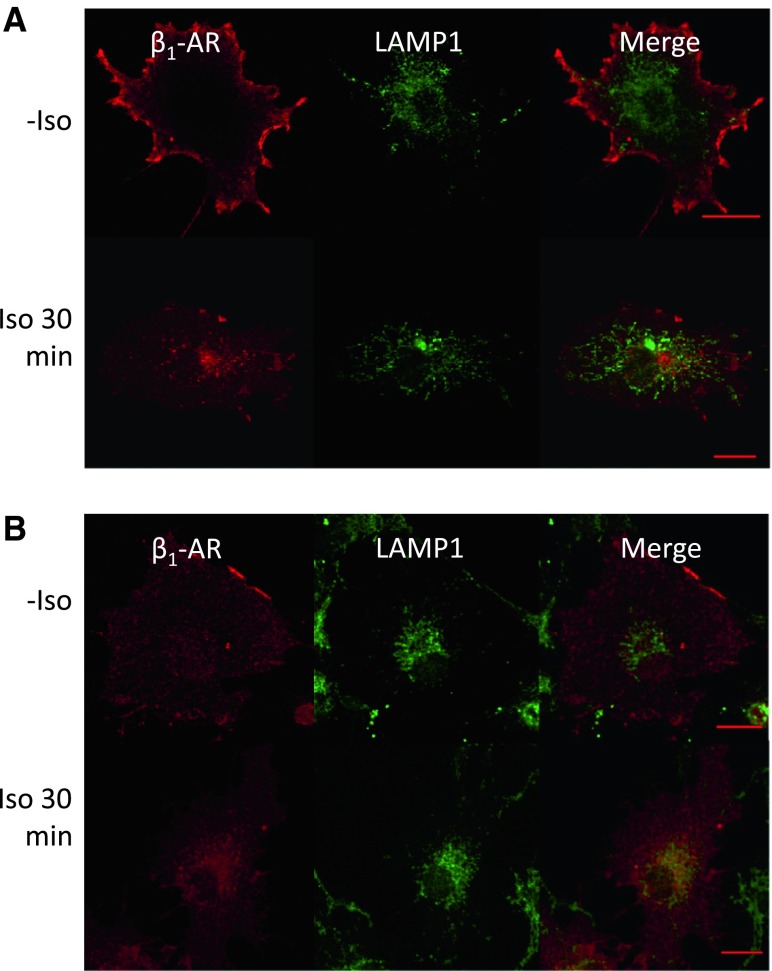
Effects of SNX27 silencing on the degradation of β_1_-AR after Iso stimulation in COS-1 cells. Empty vector (**a**) or shSNX27 (**b**) was transfected into β_1_-AR cells, and 24 h later, cells were plated onto glass coverslips. After 48 h, cells were treated without or with 10^−6^ M Iso for 30 min (−*Iso* and *Iso 30* *min*, respectively). During incubation with Iso, anti-HA Ab was added to label cell surface proteins. After incubation, cells were fixed and the internalized β_1_-AR (labeled with anti-HA Ab) was costained with anti-LAMP1 Ab. *Bar* 20 μm

Finally, the degradation of β_1_-AR was examined. Without shSNX27 treatment, most of the internalized β_1_-AR did not overlap with LAMP1, a lysosome marker, after 30 min of Iso treatment (Fig. 7a). In contrast, with shSNX27 treatment, the location of internalized β_1_-AR partly overlapped with the LAMP1-positive organelle, indicating that it was partly in the lysosome, where proteolysis takes place (Fig. 7b).

**Fig. 8:**
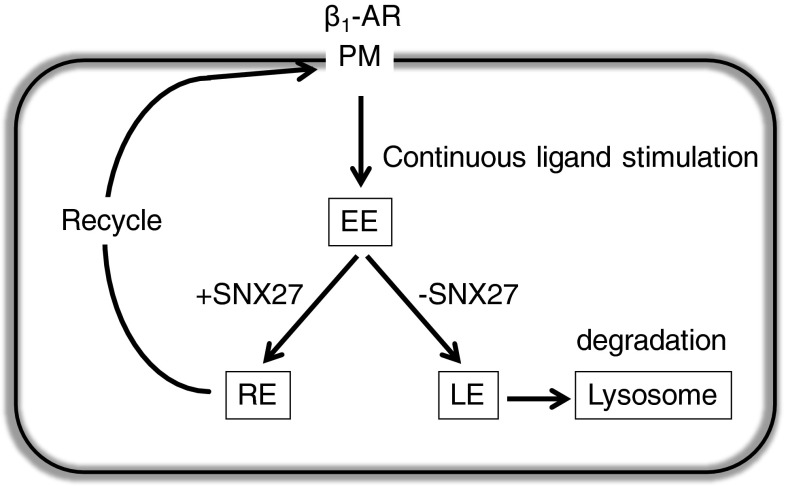
Scheme of a possible role of SNX27 in β_1_-AR intracellular traffic. Continuous ligand stimulation induces the internalization of β_1_-AR from the cell surface. β_1_-AR is first transported to EE, where SNX27 mainly localizes, and sorted into either pathway, recycling or degradation. The selection might depend on binding with SNX27. Namely, it is possible that β_1_-AR is recycled to PM through RE by binding with SNX27 in EE, while it is sorted into lysosome for degradation without binding with SNX27

We found that β_1_-AR, which has a class I PDZ-binding motif with an uncharged amino acid at the −5 position, interacts with SNX27 in EE. This is consistent with a previous report showing that the PDZ domain of SNX27 prefers acidic or uncharged residues over basic ones at the −5 position (Balana et al. 2010). As shown in Fig. 8, it is likely that internalized β_1_-AR is recycled by binding with SNX27 and degraded when such binding does not occur. In other words, the binding of β_1_-AR with SNX27 might determine its fate, recycling or degradation. It has been reported that SNX27 regulates the recycling of internalized β_2_-AR from EE/RE/LE to the PM by its interaction with retromer (Temkin et al. 2011). The complex of SNX27 with retromer seems to mediate the transport from EE/RE/LE to PM of β_2_-AR. It is possible that the SNX27/retromer complex might be involved in the PDZ-directed recycling of β_1_-AR as well. We are currently working on the involvement of retromer in β_1_-AR recycling.

β_1_-AR, but not β_2_-AR, is highly expressed in heart and regulates heart functions such as contraction and heart rate. Thus, whether β_1_-AR is recycled or degraded upon sympathetic action is essential to the recovery of sensitivity to catecholamines—particularly upon onset of heart failure, for which administration of adrenergic agents is a common intervention strategy. However, continuous stimulation of β_1_-AR often leads to desensitization, which is a common problem with this type of therapy. If we could pharmaceutically control the recycling and degradation of β_1_-AR, this problem could be circumvented. SNX27 could be a therapeutic target for controlling β_1_-AR or other proteins that have a PDZ-domain binding motif.

In conclusion, we observed that cell surface β_1_-AR is internalized into RE upon ligand stimulation and then recycled back to PM in COS-1 cells. Furthermore, we present evidence that SNX27 may be crucial in the recycling of β_1_-AR.

## Abbreviations

β_1_-AR: β_1_-Adrenergic receptor
β_2_-AR: β_2_-Adrenergic receptor
Iso: Isoproterenol
RE: Recycling endosome
SNX: Sorting nexin
TfnR: Transferrin receptor
